# Digital Platform for Pediatric Mental Health Support During Armed Conflicts: Development and Usability Study

**DOI:** 10.2196/63777

**Published:** 2024-12-26

**Authors:** Hila Segal, Arriel Benis, Shirley Saar, Iris Shachar-Lavie, Silvana Fennig

**Affiliations:** 1 Department of Child and Adolescent Psychiatry, Schneider Children's Medical Center, Petach Tikvah, Israel; 2 Shalvata Mental Health Center, Hod Hasharon, Israel; 3 Department of Digital Medical Technologies, Holon Institute of Technology, Holon, Israel; 4 Schneider Children's Medical Center, Petach Tikvah, Israel; 5 School of Medicine, Faculty of Medical and Health Sciences, Tel Aviv University, Tel Aviv, Israel; 6 Department of Behavioral Science, Israel Ruppin Academic Center, Emek Hefer, Israel

**Keywords:** pediatric mental health, digital platform, pediatricians, prevention, early intervention

## Abstract

**Background:**

The prevalence of mental health disorders among children and adolescents presents a significant public health challenge. Children exposed to armed conflicts are at a particularly high risk of developing mental health problems, necessitating prompt and robust intervention. The acute need for early intervention in these situations is well recognized, as timely support can mitigate long-term negative outcomes. Pediatricians are particularly suited to deliver such interventions due to their role as primary health care providers and their frequent contact with children and families. However, barriers such as limited training and resources often hinder their ability to effectively address these issues.

**Objectives:**

This study aimed to describe the rapid development of a digital mental health tool for community pediatricians, created in response to the urgent need for accessible resources following the October 7th terror attack in Israel. The goal was to create a comprehensive resource that addresses a wide range of emotional and behavioral challenges in children and adolescents, with a particular focus on those affected by armed conflict and significant trauma exposure. In addition, the study aimed to evaluate the platform’s usability and relevance through feedback from primary users, thereby assessing its potential for implementation in pediatric practice.

**Methods:**

A digital platform was developed using a collaborative approach that involved pediatricians and mental health professionals from various hospital clinics. The initial framework for the modules was drafted based on key emotional and behavioral issues identified through prior research. Following this, the detailed content of each module was cocreated with input from specialized mental health clinics within the hospital, ensuring comprehensive and practical guidance for community pediatricians. A focus group of 7 primary users, selected for their relevant hospital and community roles, provided feedback on the platform’s user experience, content relevance, and layout. The evaluation was conducted using a structured questionnaire complemented by qualitative comments.

**Results:**

Fifteen detailed modules were created, each providing information, including anamnesis, initial intervention strategies, parental guidance, and referral options. The focus group feedback demonstrated high satisfaction, indicating a very good user experience (mean 4.57, SD 0.53), content relevance (mean 4.71, SD 0.48), and layout suitability (mean 4.66, SD 0.52). Specific feedback highlighted the value of concise, actionable content and the inclusion of medication information. Participants expressed a strong willingness to regularly use the platform in their practice (mean 4.40, SD 0.53), suggesting its potential for broad application.

**Conclusions:**

This study demonstrates the effectiveness of a collaborative development process in creating a digital tool that addresses the mental health needs of children in crisis situations. The positive feedback from pediatricians indicated that the platform has the potential to become a valuable resource for early recognition, crisis intervention, and parental support in community pediatric settings. Future research will focus on broader implementation and assessing the platform’s impact on clinical outcomes.

## Introduction

### Mental Health Among Children and Adolescents

Mental health disorders in children and adolescents represent a significant global public health concern. According to the World Health Organization, approximately 14% of 10- to 19-year-olds experience mental health problems, yet many remain largely unrecognized and untreated [1]. Children and adolescents exposed to mass disasters, such as armed conflicts, are at higher risk of developing mental health problems. Exposure to war, conflict, and terror disrupts children’s development, leading to elevated levels of posttraumatic stress, anxiety, depression, and other behavioral and emotional reactions [2]. Beyond immediate trauma exposure, children affected by war and armed conflict endure ongoing distress due to social disruption, including home displacement, loss of loved ones, and the recruitment of a caregiver into armed group [3].

Despite the evident need for mental health support, studies indicate that a considerable proportion of children and adolescents with mental health needs do not receive the required health care and frequently endure extended waiting periods before their initial meeting with a mental health professional [4]. This delay in accessing care poses a significant obstacle, as it often leads to adverse consequences such as an increase in mental health issues [5], deterring families from seeking assistance [6], or potentially hindering treatment engagement [7]. During a sudden catastrophic event affecting the community, mental health needs may increase rapidly and exacerbate the mentioned treatment gap. Left unaddressed, these conditions can significantly impact the risk of self-harm and suicide, academic performance, social functioning, and overall well-being [8,9].

### Early Assessment

Assessment and early intervention at the onset of mental health problems have been emphasized as of great importance [8-10]. Specifically, among children affected by armed conflict, experts have pinpointed principles as “essential elements” of immediate and midterm mass trauma interventions, aimed to promote a sense of safety, calming, a sense of self-and-community efficacy, connectedness, and hope [11]. Given the close connection between emotional and behavioral problems in childhood and poorer outcomes in adulthood, early intervention reduces the duration and severity of symptoms among children and adolescents, improves long-term recovery prospects, and lessens the societal and economic burdens associated with these disorders [12].

Early intervention in mental health involves strategies across primary, secondary, and tertiary prevention levels. Primary prevention aims to prevent the onset of mental disorder, secondary prevention focuses on early identification and treatment, and tertiary prevention aims to prevent relapse and reduce the impact of ongoing mental health problems [10]. Given a severe shortage of child and adolescent mental health professionals [13], there is a growing need to adopt a setting-based approach to mental health care that integrates mental health care into community environments such as schools, community centers, and general practices. This approach increases access to quality mental health care by making mental health services more available within everyday settings [9,10].

### Pediatricians and Mental Health

Over the past decade, an increasing body of literature has highlighted the crucial role of community pediatricians in supporting early detection, intervention, and management of mental health problems in children and adolescents. By leveraging their frequent interactions with pediatric patients and familiarity with the child’s cultural and developmental context, pediatricians can significantly influence emotional and behavioral difficulties at their initial stage, often before an official diagnosis is made [14-16].

Despite the unique position of pediatricians, in the past, mental health care has typically been beyond the focus of conventional pediatric training and practice [17]. Previous studies have described that pediatricians face numerous barriers when looking to incorporate mental health tools; among them are a lack of time and insufficient confidence and knowledge in the assessment and treatment of mental health problems [18,19]. Recognizing this gap, calls for enhanced education have emphasized the importance of pediatrician competence in caring for children and adolescents with mental health needs, including skills in communication, promotion and prevention, evidence-based assessment, psychosocial intervention, and psychopharmacology [20,21].

Collaborative care model initiatives across pediatric primary care aim to enhance the integration of mental health care. These models typically involve a team-based approach where pediatricians work alongside mental health specialists to manage and treat mental health difficulties. One of the key components in collaborative mental health partnerships with pediatricians is providing mental health education [21]. State programs’ websites such as the Massachusetts Child Psychiatry Access Program [22] and Project Teach in New York [23] are such examples, providing diagnostic resources and clinical guidelines to assist in patient care. In addition, these programs offer ongoing support to pediatricians, such as real-time telephone and video consultations, and referral assistance, thereby enhancing the ability of pediatricians to address mental health difficulties effectively.. Research on collaborative care models has demonstrated substantial improvements in clinical outcomes, with studies showing increased confidence and capability among pediatricians and positive impacts on the emotional and behavioral health of children and adolescents [21].

In response to disasters, research has highlighted the need for specialized training programs for pediatric primary care providers to address the impact of these events on community mental health [24-26]. For example, in response to Superstorm Sandy in New York, the Pediatric Disaster Mental Health Intervention was developed to equip pediatric health care providers with the psychosocial skills necessary for effectively caring for children in disaster zones. This program included focused, in-person training that equipped providers with the tools to manage mental health issues arising from such events [24]. On the digital side, tools such as PsySTART have been designed for rapid mental health assessment and management, designed to assist health care professionals in assessing trauma exposure, identifying high-risk individuals for timely interventions, and aiding in the efficient allocation of mental health resources based on real-time data [25,26].

### Previous Work

Prior research by the authors of this paper evaluated the feasibility of the Hebrew parent version of the Pediatric Symptom Checklist (PSC-17) to enhance early mental health screening in pediatric settings [27]. This study involved 274 parents in outpatient clinics and included collaborative sessions with pediatricians and mental health professionals to improve intervention strategies and communication techniques in primary care setting. These discussions identified key pediatric mental health concerns, which informed the development of the 15 modules used in the current digital platform.

### The Current Context

On October 7, 2023, Israel faced an unprecedented attack by Hamas terror organization [28], one of the deadliest terrorist attacks in recent decades [29]. This event, as in other mass traumas [30], significantly increased the prevalence and severity of mental distress across Israel’s civilian community [31,32], highlighting an urgent need for effective, immediate mental health interventions.

In response, this study examines an innovative digital platform designed to support primary care pediatricians in delivering large-scale mental health services during times of crises. The platform aims to facilitate early intervention, support parental psychoeducation, and strengthen the collaborative care network among pediatricians, mental health professionals, and families.

### Aims and Objectives

We aspire to continue the paradigm shift in primary pediatric care, recognizing mental health as an inseparable component of the child’s overall well-being. Our primary objectives are (1) to demonstrate the feasibility of a digital mental health platform specifically designed to address the urgent needs arising from crisis situations, facilitating early intervention and timely mental health care and (2) to raise awareness, instill responsibility, and increase knowledge among pediatricians in assessing and treating emotional and behavioral concerns.

## Methods

### The Platform

Drawing upon the foundational work conducted in previous research, existing knowledge was adapted to develop a mental health digital platform tailored to the specific needs of pediatricians. The platform was designed to enhance pediatricians’ expertise, competence, accountability, and confidence in identifying, evaluating, and initiating early interventions for mental health concerns within pediatric primary care. The platform includes 15 modules, each focused on addressing the common emotional and behavioral challenges identified as critical in prior research, essential for effective pediatric mental health care in the context of the recent trauma.

The digital platform was developed at Schneider Children’s Medical Center of Israel through a collaboration process between the project team members, including primary care pediatricians and mental health professionals.

Experienced pediatricians in the project team provided critical insights into the workflow design and suggested the structure for the modules, including sections on anamnesis, parental psychoeducation, intervention strategies, and “red flags” necessitating mental health referral. This foundational structure was then handed to specialized clinics within the psychological department at the hospital to populate the content with detailed, evidence-based information. Each psychological clinic contributed based on their expertise; for instance, the eating disorders clinic developed the content for the module on “changes in eating behavior,” and the anxiety clinic contributed to the module on “sleeping problems.”

Once the initial drafts of all modules were completed, the project lead team conducted a thorough review to ensure consistency and comprehensiveness. This collaborative effort was essential in adapting the information for the digital platform, ensuring that it was user-friendly and aligned with clinical best practices.

This detailed and systematic development process, which involved numerous professionals from Israel’s largest pediatric hospital, underscores the platform’s credibility and practical use in addressing pediatric mental health issues.

### Focus Group

A focus group of 7 pediatricians was gathered following the platform design in order to collect prompt feedback and improvement suggestions. The recruited pediatricians were all required to have completed their residency, ensuring that they had sufficient experience and expertise to provide informed feedback. They were selected based on their roles as pediatricians working both in hospital settings and within general pediatric practices in the community. This dual experience was crucial, as it ensured that the participants were familiar with the diverse challenges faced by pediatricians in different health care environments. Importantly, the participants were independent of the platform’s design process and the project team, ensuring unbiased and objective feedback.

Pediatricians were identified through professional networks and affiliations with community clinics and the hospital. Fifteen pediatricians were contacted to participate in the evaluation of the platform, of whom 7 (46.6%) agreed to participate. The recruitment enquiry provided details about the study’s objectives, the scope of the platform being evaluated, and the voluntary aspect of participation.

Participants were instructed to freely browse the digital platform to familiarize themselves with its overall structure and content. Following this exploration, each participant was asked to choose a single module that they found most relevant or interesting to their practice for a more detailed evaluation.

A 12-item scale was constructed; 11 items were rated on a 1‐5 Likert scale, with higher ratings reflecting greater satisfaction. One additional item allowed participants to provide qualitative feedback for suggestions on platform improvements. The key measures included in the test were the General Satisfaction Domain and the Specific Content Satisfaction Domain.

In the General Satisfaction Domain, 4 questions addressed the general use of the website and included questions such as “How relevant is the content in this website to your work with children?” and “How would you rate the user experience?” The items in this domain are discrete; therefore, there is no rationale for calculating internal consistency.

In the Specific Content Satisfaction Domain, 7 additional questions addressed a specific content page of choice (eg, trouble sleeping). Items included questions such as “How useful was this topic for you?” and “How accurate is the layout of the page to your clinical work?” The questions in this domain were averaged to an overall satisfaction score. Overall specific content satisfaction domain variable demonstrated an acceptable internal consistency (Cronbach α=0.77).

### Ethical Considerations

This study was conducted in accordance with the Declaration of Helsinki. This study was granted exemption by the Rabin Medical Center Helsinki committee (exemption letter available upon request). According to institutional regulations, ethics approval was not required as the study involved the development and evaluation of an educational tool for health care professionals. All participants were adults and provided informed consent before participation, with an understanding of the study’s objectives and their right to withdraw at any time. No compensation was provided to participants. All collected data were anonymized and securely stored on an institutional computer managed by the principal investigator (HS) to ensure confidentiality and data protection.

### Statistical Analyses

Descriptive analyses were conducted with the focus group qualitative and quantitative data. Cronbach α was calculated to test reliability. Microsoft Excel 2023 and SPSS software (version 26; IBM) were used in the data analysis.

## Results

### The Platform

**Figure 1.**
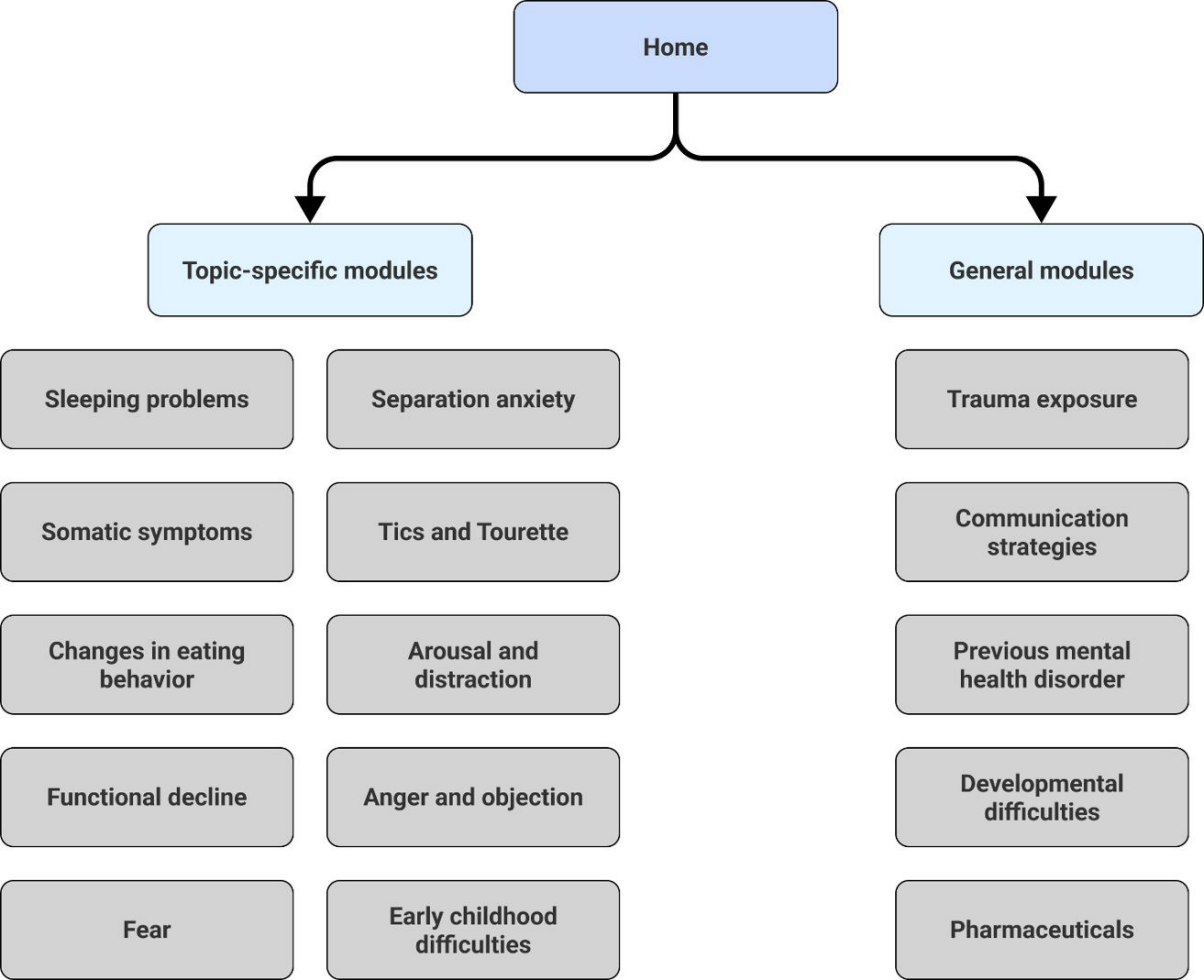
Architecture of the digital platform for pediatric mental health assessment and intervention.

The digital platform comprises 15 modules, divided into general and topic-specific modules (Figure 1; Multimedia Appendix 1). Five general modules cover foundational topics including information about trauma exposure, communication strategies during heightened time of mental needs, children with previous mental health disorder, children with developmental difficulties, and common psychiatric pharmaceuticals including recommended dosage and use.

Ten topic-specific modules delve into common emotional and behavioral challenges, prevalent among children and adolescents in primary care setting. These include presenting symptoms such as changes in eating behavior, sleeping problems, and fear and anxiety.

Each module includes information about common concerns that could be exhibited, detailed anamnesis, parental psychoeducation, intervention strategies, and “red flags” necessitating mental health referral. A highlighted description of the modules, the categories, and the corresponding items is shown in Table 1.

**Table 1.** Emotional and behavioral modules developed for the digital platform, detailing the focus areas and key items for pediatric mental health assessment.

Module	Common concerns	Anamnesis	Psychoeducation	Intervention strategies	Red flags
Sleeping problems	Difficulty falling asleep independently; desire to sleep in parents’ bed; disturbing thoughts before going to sleep (eg, about death and worry); reversal of sleep patterns; nightmares and night terrors; and sleeping at opposite times (sleeping during the day and awake at night).	Were there difficulties before the war? Does the child have a mental health history? Is the child receiving medication? If so, is the medication taken in the recommended dose? Has there been a change in the family dynamic (eg, a family member recruited, injured, or passed away)? What specific issues are present (difficulty falling asleep, waking up in the middle of the night, nightmares)? Are there night sweats? How often?	Attention to the emotional state of the parent; legitimacy and empathy; encourage sleep routine; explain ways to give the child a sense of control over nightmares; and address regression to previous sleep patterns.	Psychoeducation; active listening; legitimization and normalization; empowerment of the child’s strengths; and collaborative problem-solving.	Difficulty sleeping at high intensity without gradual improvement over time; distress and difficulties in other areas of functioning (school avoidance, social withdrawal, bed wetting, etc).
Somatic symptoms	Abdominal pain; headaches; pain in different parts of the body; change in body mobility; weakness; dizziness; difficulty breathing; and feeling that something is stuck in the throat.	What type of physical pain does the child experience? At what intensity and frequency? When did the behavioral change begin? What is the level of parental concern regarding these changes? Is the pain consistent throughout the day or does it appear near certain events (alarms, watching the news, and sudden noises)? Has the child suffered from an organic physical illness recently? What is the family’s reaction to the child’s physical complaints?	Identify and explain parental reactions that reinforce symptoms and increase psychosomatic pain (eg, allowing the child to avoid school, the child entering the parent’s bed); verbal expression of difficulties can reduce physical distress; soothing, especially in times when there are no physical complaints; and decrease behavioral avoidance.	Psychoeducation; active listening; legitimization and normalization (“it hurts you”); empowerment of the child’s strengths; and collaborative problem-solving. With young children, keep the discourse concrete about the pain (ice for the leg, warm pillow for the tummy). With teenagers, address pain as an expression of stress and anxiety.	Physical symptoms of high intensity without gradual improvement over time.
Changes in eating behavior	Limited food intake (quantity and variety); Significant weight loss in a short period; intense fear of weight gain; obsessive preoccupation with food and calories; episodes of eating large amounts of food in a short period; feeling a lack of control while eating; compensatory behaviors: vomiting, increased physical exercise, and use of laxatives or enemas; avoidance of eating with others; and bad mood.	When did the changes in eating start? What caused the weight loss? What are the child’s satisfaction goals and future plans? What is the daily food intake? Have there been changes in eating habits (including veganism or vegetarianism)? Is there excessive exercise? How does the child feel physically? Are there symptoms of weakness or dizziness? Is menstruation regular?	Focus on messages about body and mind health rather than appearance; maintain an assertive stance regarding health and emphasize problems arising from improper eating; parental attention to systematic or increasing changes; and supervision over eating, physical activity, vomiting, and laxative use.	Psychoeducation; physical assessment: inquiry about menstruation, weight, height, BMI, blood pressure, pulse, ECG, and blood tests (including electrolytes). Schedule monitoring appointments.	Significant eating difficulties without gradual improvement over time; drastic weight loss in a short period (even if BMI is normal); physical symptoms indicating distress; and cessation of growth and development.
Functional decline	Bad mood; reduced interest or enjoyment in activities; school refusal; social withdrawal; difficulties in relationships; learning difficulties; reduced ability to concentrate or think clearly; outbursts of crying and rage; irritability; and impulsiveness.	Is the child sad? If so, at what intensity and frequency? Are there changes in appetite, sleep, school attendance, or socializing with friends? When did the behavioral changes begin and what is their intensity? What helps calm the child’s reactions?	Encourage empathy toward the child’s difficulties; promote a return to routine and functioning in small steps, routine creates a sense of security and stability; collaborate with the child to develop an adapted and creative plan to address the difficulties. For example, accompany the child to school, start with short attendance days, and gradually increase; go for evening walks in the neighborhood.	Psychoeducation; active listening; legitimization and normalization; empowerment of the child’s strengths; collaborative problem-solving.	Various symptoms at high intensity without gradual improvement over time; distress and difficulties in various areas of functioning (school refusal, social withdrawal, difficulties in relationships, etc); and in case of suicidal statements or violent behavior—refer for immediate psychiatric evaluation.
Fear	Emotional difficulties: restlessness, despair, worry, helplessness, and constant crying; behavioral difficulties: sleeping issues, fear of separation from parents, and school avoidance; cognitive difficulties: disturbing thoughts, concentration and memory issues, and worrying about negative events; and physical difficulties: abdominal pain, nausea, vomiting, diarrhea, rapid pulse, and feeling of suffocation.	Is the child receiving medication? If so, is it taken in the recommended dose? Has there been a change in the family composition (eg, a family member recruited, injured, or passed away)? What fears does the child experience? At what frequency and intensity? What physiological reactions accompany the fears? How long do they last? What helps?	Recognize that fear and anxiety reactions are natural during this period; encourage parents to be patient, empathetic, and attentive to the child’s difficulties; encourage activities that promote calming; and maintain a routine.	Psychoeducation; active listening; legitimization and normalization; empowerment of the child’s strengths; and collaborative problem-solving.	Symptoms of high intensity over time without gradual improvement; distress and difficulties in other areas of functioning (school avoidance, social withdrawal, etc).
Separation anxiety	Excessive concern for parents; physical attachment to parents; prolonged crying without relief after separation; anger; disturbing thoughts; sleep difficulties; and difficulty forming attachments with other figures (eg, kindergarten teacher).	What happens during separation from parents? How long does it take for the child to calm down? What helps the child during separation? What has helped in the past? Are there accompanying anxiety symptoms (constant thoughts, fears, and sleep difficulties)? Are there accompanying depressive symptoms (low mood, difficulty functioning, and withdrawal)?	Recognize that separation anxiety is usually a natural reaction to stress and anxiety; encourage parents to be patient, empathetic, and attentive to the child’s difficulties; promote age-appropriate behavior and independence in small, gradual steps; use coping strategies such as reading stories about separation, providing a transition object (parent’s clothes, teddy bear, and blanket), and establishing a separation routine (keyword, special high-five); and with older children, schedule regular phone or video call times throughout the day.	Psychoeducation; active listening; legitimization and normalization; empowerment of the child’s strengths; and collaborative problem-solving.	Significant separation difficulties without gradual improvement over time; distress and difficulties in other areas of functioning (school avoidance, social withdrawal, etc).
Tics and Tourette	Common movement tics: eye blinking, looking away, facial twitching, head tilting, sudden limb movements, neck or limb stretching, and jumping; common vocal tics: throat clearing, humming, nasal pulling, annoying coughing, pronouncing syllables, words, and repeating sentences.	When did the tics start? Have new behaviors or additional symptoms appeared with the tics? What motor or vocal tics are present? Ask the patient to demonstrate. Is there a feeling that precedes the tic? Do the tics interfere with daily functioning? Do parents comment on the child’s tics?	Explain that tics are not dangerous and do not cause harm; emphasize that the child does not control the tics, so they should not be warned or punished; stress and anxiety can worsen tics; excessive attention to tics and reinforcement of behaviors can make them worse; trying to hide tics can cause shame and guilt; and talk openly about tics in an age-appropriate manner.	Psychoeducation, active listening, legitimization and normalization, empowerment of the child’s strengths, collaborative problem-solving; and medication.	Pay special attention to differential diagnosis, including the possibility of another neurological disorder; tics that start at a relatively late age with a dramatic appearance may be pseudotics (functional tic-like behaviors); multiple, powerful, and painful tics that interfere with daily functioning and well-being; complex tics involving self-harm; and significant accompanying psychiatric disorders.
Arousal and distraction	Difficulty maintaining attention for long periods; easily distracted; difficulty having conversations; physical arousal; restlessness; nervousness; difficulty sitting still; and high mobility.	Is the child receiving medication? If so, is it taken in the recommended dose? What kind of difficulties is the child experiencing? In which settings are they mainly expressed (school, home, or both)? What is the intensity of the symptoms? When did the behavioral changes begin? What calms the child? What has worked in the past?	Emphasize the importance of parental security and stability; recognize that reactions such as restlessness and distraction are often natural responses to stress rather than indicators of ADHD^a^; establish daily routines such as morning routines and school attendance; encourage independence in activities such as personal hygiene and meal preparation; and provide opportunities for energy release through outdoor play, sports, or jogging in the neighborhood.	Psychoeducation; active listening; legitimization, and normalization; empowerment of the child’s strengths; and collaborative problem-solving.	Symptoms of arousal and distraction of high intensity that did not exist before the war or have increased significantly; symptoms do not improve gradually over time; and violence.
Outbursts of anger and objections	Tempers and tantrums; yelling; prolonged crying; impatience; reluctance to cooperate; and breaking rules and norms.	Is the child receiving medication? If so, is it taken in the recommended dose? What kind of difficulties is the child experiencing? In which settings are they mainly expressed (school, home, or both)? What is the intensity of the symptoms? When did the behavioral changes begin? Are there changes in appetite, sleep, school attendance, and socializing with friends?	Understand that outbursts of anger and resistance can signal underlying distress and a desire to assert control; recognize that during intense anxiety, children may experience emotional meltdowns; acknowledge that reactions to stress are common and natural, including resistance, anger outbursts, and tears; and provide outlets for energy release through outdoor play or sports activities.	Psychoeducation; active listening; legitimization and normalization; empowerment of the child’s strengths; and collaborative problem-solving.	Symptoms of high-intensity resistance and tantrums that did not exist before the war or have increased significantly; symptoms do not improve gradually over time; and violence.
Early childhood difficulties	Emotional reactions: fear and anxiety, sadness, frustration, anger, and tantrums; physical reactions: stomach aches, headaches; cognitive responses: difficulties with attention and concentration; and behavioral reactions: withdrawal, parental attachment, and regression (eg, crawling into the parent’s bed at night, difficulty sleeping, bedwetting, and returning to the pacifier).	Are there other small children at home? Has there been a change in the family composition (eg, a family member recruited, injured, or passed away)? What is the level of parental anxiety? Are there changes in appetite, sleep, or kindergarten attendance? How concerned is the parent regarding these changes? What calms the child?	Recognize the parent as the primary caregiver responsible for the child’s support and care; if the parent shows signs of distress, seek assistance from another parent or family member; encourage the child to respond to challenges appropriately for their age and developmental stage; promote empathy and inclusion; encourage joint calming activities for the parent and child; and avoid avoidance behaviors as they may not alleviate distress effectively.	Psychoeducation; active listening; legitimization and normalization; empowerment of the child’s strengths; emphasis on parental strengths; and collaborative problem-solving.	No improvement or relief in distress over time; reactions include severe obstacles that significantly affect the normal development of the child and the parent.

a ADHD: attention deficit hyperactivity disorder.

### Focus Group

The focus group consisted of 7 pediatricians working both in the hospital and in Clalit Health Services community clinics in central Israel: 57.1% (n=4) were female, the mean age was 36.57 (SD 3.69) years, and the average work experience in pediatric care was 6.29 (SD 1.38) years. Participants provided individual feedback through structured questionnaires, which yielded quantitative data on user experience, content relevance, and platform usability. Additional qualitative comments were also gathered, offering further insights into specific areas of the platform. Their feedback indicated a very good user experience (mean 4.57, SD 0.53), relevant content (mean 4.71, SD 0.48), and professionally suitable content layout (mean 4.66, SD 0.52). The pediatricians reported that they would regularly use the platform (mean 4.40, SD 0.53). In the specific content domain, a subset of the 15 available modules was reviewed in depth. The specific modules chosen by the pediatricians included “sleeping difficulties,” “Tics and Tourrete,” “previous mental health problems,” “changes in eating behavior” and “early childhood difficulties.” Overall great satisfaction score was reported (mean 4.66, SD 0.30).

Participants commented on the platform in general, “I liked the concept, the information is relevant and easy to use”; “important platform for clinical work”; and “great idea for community pediatricians.” They also provided valuable suggestions for further improvement, such as shortening the text to align with time constraint, including more detailed medication information, and expanding the platform’s availability in English and Arabic. These suggestions were incorporated into the platform refinement and will be included in the updated versions.

## Discussion

### Principal Results

Current global conflicts often involve violence and attacks directed toward civilian communities, instilling fear, danger, and insecurity. Among the most vulnerable are children and young people, who face immediate threats and disruptions to their daily lives [2]. This study focuses on the collaborative efforts of pediatricians, mental health professionals, and digital experts to develop a platform aimed to improve the accessibility and delivery of mental health services during crises. The development process of the platform is detailed in this study, along with the results from a focus group of primary users. The findings indicate that the platform has significant potential to enhance the ability of pediatricians to assess and manage mental health concerns following traumatic events.

The platform is specifically tailored to the primary care environment, focusing on early emotional and behavioral problems rather than diagnosis labels. This approach empowers pediatricians to intervene early, potentially preventing symptom escalation and reducing the burden of mental illness on affected individuals and their families [9,10,14]. In addition, each of the 15 modules is designed to accommodate pediatricians’ time constraint, offering concise, actionable guidance that can be accessed quickly for guidance during patient visits or explored in more depth when time allows. This flexibility ensures the platform’s use regardless of the user’s time limitation.

The platform also emphasizes parental psychoeducation, recognizing the crucial role of parental support and familial cohesion in children’s mental health [33]. By fostering effective communication between pediatricians, patients, and parents, the platform aims to build strong therapeutic alliances and enhance patient care and support [14,33].

Developed through a collaborative effort involving pediatricians and mental health specialists, the platform ensures content that is both clinically accurate and practical for primary care use. A multidisciplinary approach is particularly significant, given the role of inadequate public understanding of mental health in contributing to the treatment gap, hindering help-seeking behaviors, and maintaining stigma [34]. Many mental health platforms publicly available offer potential solutions with a wide range of functionalities, including education, screening, and self-management programs. However, many fall short of delivering accurate, evidence-based information [35]. This challenge further reinforces misconceptions and misinformation among individuals. Thus, there is a significant opportunity to provide quality comprehensive education and simple pathways for intervention in mental health, as facilitated by pediatricians through the platform.

Launched during a national crisis, the platform met an immediate and critical need for mental health support amidst heightened stress. It addresses specific challenges faced by children exposed to trauma, such as war and conflict, natural calamities, or acts of terrorism, providing targeted interventions for both direct and indirect impact of mass trauma. In light of the shortage of child and adolescent mental health professionals [13], especially during mass trauma situations, this initiative underscores the importance of equipping pediatricians with the knowledge and tools to provide timely mental health care. Moving forward, the platform will be publicly available and continuously improved through feedback from its primary users. This ongoing development will ensure that the platform remains relevant and effective, better supporting pediatricians in real-world settings.

### Limitations

The focus group, while using a small and specific sample, was assembled to rapidly address the urgent need for platform distribution. While the study was designed as a focus group evaluation, logistical constraints and the need for rapid feedback necessitated the use of individual questionnaires. This method allowed participants to provide independent feedback but limited the opportunity for interactive dialogue. Furthermore, while the focus group provided an overall satisfaction rating for the platform, participants conducted an in-depth evaluation of only one module of their choice. Therefore, only a subset of modules was evaluated during this initial review. In addition, participants did not provide feedback on the time it took to engage with the platform. As a result, while the platform was designed with time efficiency in mind, the actual time investment required from pediatricians was not formally assessed, leaving an important aspect of the user experience unexplored. Future research should aim for a comprehensive evaluation study with a large sample size and a rigorous research design to further validate the platform’s effectiveness. This would ideally include interactive discussions to capture a broader range of feedback and insights.

While the platform offers valuable self-guided tools and resources to support pediatricians in addressing mental health concerns, it does not currently include features such as real-time psychiatric hotline, consultation, or direct patient evaluation. These elements are considered in future iterations to further connect primary care with child psychiatry and enhance the platform’s support for users.

### Conclusions

Mass trauma events highlight the urgent need for swift and comprehensive community interventions in pediatric mental health. Recognizing the critical time frame from initial symptom manifestation to disorder onset, the rapid development and deployment of a digital mental health tool for primary pediatric care represents a significant advancement in providing immediate support to young individuals. This initiative demonstrates the feasibility of a responsive and targeted digital mental health platform during periods of heightened need, emphasizing the essential role of pediatricians in delivering timely and proactive care, both in routine situations and during crises.

## Supplementary material

10.2196/63777 Multimedia Appendix 1 An overview of the homepage of the digital platform.

## Abbreviations

PSC-17: Pediatric Symptom Checklist
