# Supplementary figures and images for: Digital Platform for Pediatric Mental Health Support During Armed Conflicts: Development and Usability Study
Source: JMIR Form Res. 2024 Dec 26;8:e63777. doi: 10.2196/63777 (PMC11695804; doi:10.2196/63777)

Multimedia Appendix: An overview of the homepage of the digital platform.


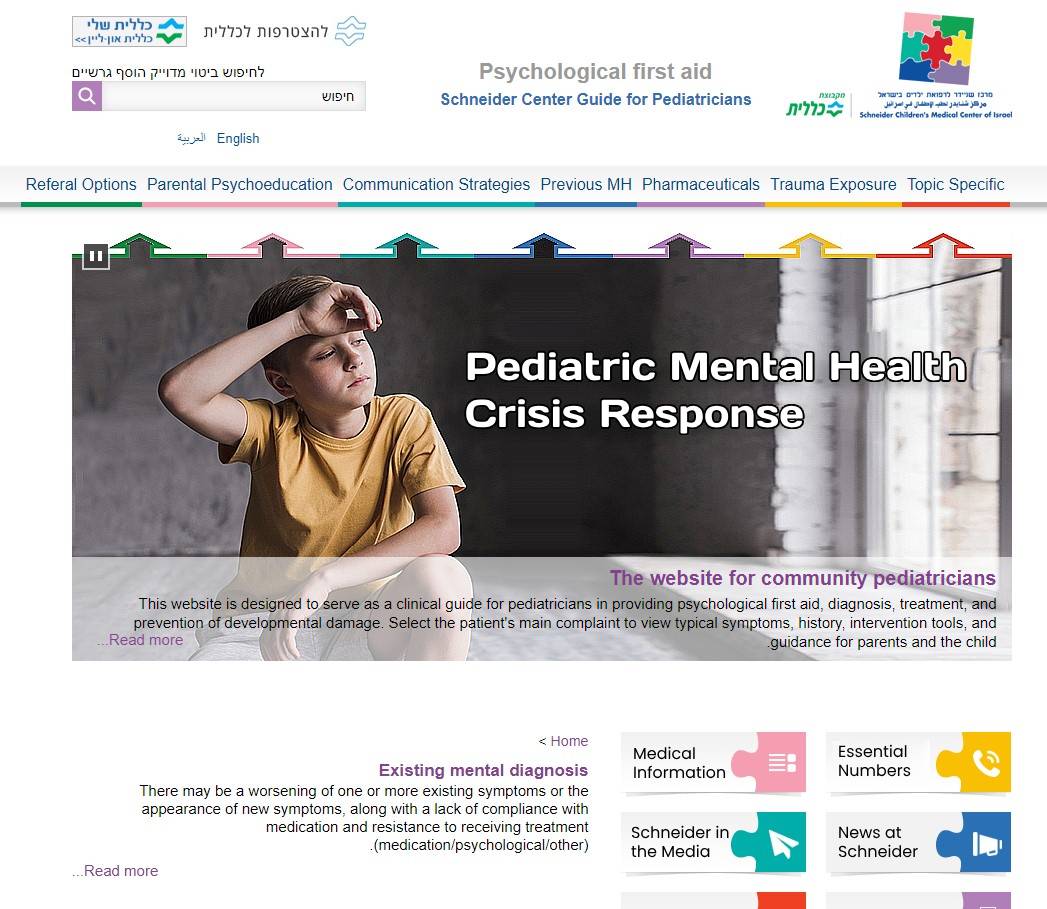

Supplement: Multimedia Appendix 1 [file formative-v8-e63777-s001.docx]
